# Supplementary material for: How socioeconomically disadvantaged people access, understand, appraise, and apply health information: A qualitative study exploring health literacy skills
Source: PLoS One. 2023 Aug 9;18(8):e0288381. doi: 10.1371/journal.pone.0288381 (PMC10411818; doi:10.1371/journal.pone.0288381)
Supplement: S1 Table — (DOCX) [file pone.0288381.s001.docx]

**S1 Table: Additional participant quotations**

| **Themes** | **Sub-themes** | **Categories** | **Additional quotes** |
| --- | --- | --- | --- |
| Financial insecurity triggers the need for health information | Recognizing the need for information to prevent unnecessary illness and save money | Being confronted with a health promotion message | *"I learned by watching TV, the advertisement "Eat 5 fruits and vegetables a day". I said to myself, "Here! 5 fruits and vegetables a day. But I don't do that at all!" I saw how I was eating, and I wasn't doing well. I was going to ruin my health." (Participant 11)* |
|  | Finding the best way to treat the disease while maintaining financial stability | Being responsible for one’s own health | *“I still try to inform myself [about my high blood pressure problem]; I don’t want to be irresponsible!” (Participant 7)* |
| Pathway 1: Physicians as ideal (but expensive) interlocutors | Physicians as the main reference points for getting health information | The GP is the only identified source of health information | *“I don’t know where to go to get health information… So the only way for me to get this information is through my GP.” (Participant 8)* |
|  | Difficulty of obtaining health information due to limited access to a physician | The financial cost of a medical consultation cannot be afforded | *"If I go to the doctor now, what do I do with the bill, how am I going to pay? But as soon as I have some money saved up, I'll go to the doctor." (Participant 4)* |
|  |  | Identifying alternative sources of information | *“But when I know I can’t pay, that I don’t have any money, no cash on hand, I ask my [female] friends first. I’ve also asked a client who often comes to get her hair done.” (Participant 4)* |
|  | Insufficient information due to being disregarded or overly brief consultations | Overly brief consultations | *"It's true that doctors have a lot of work to do. Sometimes, some doctors, they have no patience, they don't take the time [to give information]. The time is running out... That's how life is, that's the system.... " (Participant 12)* |
|  |  | Feeling disregarded | *"From the doctors’ perspective, they are the only ones with a degree, they are the ones who know! We [the patients] know nothing, and they know everything! I think that doctors should listen more to people. Can't they see we're human? That we have not studied? We don't know anything, it's true... But we probably have other values..." (Participant 9)* |
|  | Medical jargon makes information hard to understand | Being confronted with unfamiliar medical jargon | *"It depends... Sometimes, yes, it's difficult [to understand the doctor's explanations] ... For people who are not from the medical field, it's sometimes a bit difficult... " (Participant 7)* |
|  | Health information provided by GPs is not questioned | Conflicting information from different health professionals | *"The doctor had prescribed a medicine, it was antibiotics, and then he had told me it was to put through the vagina. To the drugstore, the pharmacist told me: "You have to swallow it". I said: "No, he [the doctor] said in the vagina". "Madam, I tell you!" (with an authoritative tone). Boo... I was upset! I said to myself: "Now what do I do? What should I do?" I called the doctor back..." (Participant 4)* |
|  | Striving to apply the medical treatment | Too expensive medicines | *"I ordered this drug in France because the problem is the price. In Switzerland, you have 30 tablets for past 25 or 30 francs a box. In France, I have 180 tablets for 20 euros." (Participant 4)* |
| Pathway 2: The internet as a suboptimal alternative | The internet as a tool to overcome barriers related to medical consultations | Internet’s advantages | *"Compared to the doctor, well, the internet is easier and faster. And especially free. That's the advantage." (Participant 1)* |
|  | The internet as a resource for day-to-day health issues | Seeking information for everyday health problems | *"If, for example, I have a stomachache, I do a Google search. There I find that a chamomile tea helps and then... actually, it's mostly for small boo-boos." (Participant 1)* |
|  | Hard-to-understand online information related to medical terminology and inadequately written information formats | Difficult and unfamiliar words | *“Sometimes it’s difficult on the internet because there are words that I don’t understand.” (Participant 4)* |
|  | Online health information is analyzed critically and viewed with suspicion | Not all information is trustworthy on the internet | *“We know that the internet is a trash can. A very big trash can! When you’re not on trusted websites, it’s garbage!” (Participant 3)* |
|  | Online health information is selected on the basis of subjective and objective criteria | Information selection based on financial and material means | *"If, assuming I find information on Google that says I need to do something but I can't afford it, or I don't have the ingredients at home, well, I'll go to another piece of information where there may be other solutions." (Participant 1)* |
|  | Using simple, easy-to-implement online health information | Implementation of small tricks at no cost | *"For example, my kids, there's one who has diarrhea all the time. I found in the computer [on the internet]. And it was the cornstarch, the same you use for cakes... So it's noted, you have to give 15 minutes before each meal, mixed with water. It's not hard to remember, it's not hard to do that!" (Participant 9)* |
| Pathway 3: Relatives as a default resource | Trusted relatives are asked for health information | Social network members familiar with the health topic | *“I ask people around me, but people who...I'm not going to talk to the milkmaid about it, I mean...I will talk to people I know, who know the field or the subject.” (Participant 10)* |
|  |  | Confidentiality | *"I first ask my girlfriends for information. Not all the girlfriends, eh! Because I like to be discreet, I don't like people who talk about other people's things that don't concern them, I don't like..." (Participant 4)* |
|  | Trusted relatives have no answer | Relatives have the same difficulties with health information | *"At first, I asked my friends for health information. Then I saw that it annoyed them. And then afterwards I regretted it. No, because they were helpless. The big problem is that the people around you are helpless, they don't know what to do. " (Participant 3)* |
