# Supplementary material for: How socioeconomically disadvantaged people access, understand, appraise, and apply health information: A qualitative study exploring health literacy skills
Source: PLoS One. 2023 Aug 9;18(8):e0288381. doi: 10.1371/journal.pone.0288381 (PMC10411818; doi:10.1371/journal.pone.0288381)
Supplement: Attachment — Submitted filename: Response to reviewers.docx [file pone.0288381.s004.docx]

Response to reviewers

Dear editor,

We thank the reviewers for their comments. We improved the manuscript based on the reviewers’ comments. Specifically, we have amended the text of the manuscript as outlined below.

Reviewer # 2:

1. **The manuscript is still very long and you did not included any new reference from 2022 or 2023.**

We thank the reviewer for this comment. To take it into account, we have reduced the results and the discussion sections. Regarding references, they have been updated, and references from 2022 and 2023 have been added. In addition, the references list has been reduced.

Reviewer # 3:

1. **One comment to note - the data of this study was collected between April and June 2017. Given that this is now April 2023, this needs acknowledged as a limitation of this study.**

We thank the reviewer for pointing out this limitation. This observation has been added to the limitations of our study on page 41.

Reviewer # 4:

1. **It may also be useful to add the age range of participants to the Abstract.**

As asked, the age range of our participants has been added to the abstract.

2. **The authors could do more to address Reviewer 2 (Comment 4), particularly by shortening the discussion such that the main findings are summarised rather than being described again.**

We acknowledge that the manuscript was still very long. To take this comment into account, we have revised and reduced the discussion section as much as possible.

3. **Typographical errors: Page 11: “All the transcriptions were check entirely for accuracy” should be “All the transcriptions were checked entirely for accuracy”.**

We thank the reviewer for this careful reading. We apologize for this typing error and have accordingly corrected it.

4. **I find the wording of the first theme slightly awkward: “Getting informed for not worsening a precarious financial situation”. I encourage the authors to consider slight revisions for clarity.**

We thank the reviewer for this interesting comment. To take it into account, the first theme has been modified on page 17 for better clarity.

5. **Data was collected in 2017. Much has happened since then, particularly COVID which changed the way that many of us receive health information. I encourage the authors to reflect on how changes since then might have impacted the data collected, for example, in the Strengths and Limitations section of the manuscript.**

We thank the reviewer for pointing out this limitation. The impact of the COVID-19 pandemics on health literacy skills has been added to the limitations of our study on page 41.

We thank you again for the work done on this manuscript and remain at your disposal for any further information.

The authors.
