# Supplementary material for: How socioeconomically disadvantaged people access, understand, appraise, and apply health information: A qualitative study exploring health literacy skills
Source: PLoS One. 2023 Aug 9;18(8):e0288381. doi: 10.1371/journal.pone.0288381 (PMC10411818; doi:10.1371/journal.pone.0288381)
Supplement: Attachment — Submitted filename: Reviewer comments LP March 20223.docx [file pone.0288381.s003.docx]

All comments and suggestions were responded based on my comments and changes were made to the manuscript. Although the number of pages has been significantly reduced, it now has 47 pages and 15 more pages of references (there are 120 references and only 19 of which are from the last five years and the most recent from 2021). From point of view, the content is fine but It is up to the editors to accept the article with 47 pages plus 15 references.
